# Making Statistical Sense of the Molnupiravir MOVe-OUT Clinical Trial

**DOI:** 10.4269/ajtmh.21-1339

**Published:** 2022-03-11

**Authors:** Kristian Thorlund, Kyle Sheldrick, Gideon Meyerowitz-Katz, Sonal Singh, Andrew Hill

**Affiliations:** ^1^ Department of Health Research Methods, Evidence & Impact, McMaster University, Hamilton, Ontario, Canada;; ^2^ University of New South Wales, Kensington, Australia;; ^3^ School of Health and Society, University of Wollongong, New South Wales, Australia;; ^4^ Department of Family Medicine and Community Health, UMass Chan Medical School, Worcester, Massachusetts;; ^5^ Department of Pharmacology and Therapeutics, University of Liverpool, Liverpool, United Kingdom

## Abstract

Oral therapies for the early treatment of COVID-19 may prevent disease progression and health system overcrowding. A new oral therapeutic named molnupiravir has been promoted as providing an approximately 50% reduction in death or the need for hospitalization. The clinical trial evaluating this drug was stopped early at the recommendation of the Data Safety and Monitoring Board after approximately 50% of the sample had been recruited. At the point of discontinuing the trial, approximately 90% of the planned sample had been recruited and had available follow-up data accessible. We discuss issues about the study conduct, analysis, and interpretation, including 1) the authors and sponsors presented the interim analysis as the primary analysis; 2) communication between sponsors and the Data Safety and Monitoring Board was insufficient; 3) the treatment effects reverse when examining only the post-interim analysis population, and are substantially attenuated when examining the full data; 4) the choice of primary analysis is incorrect; 5) analysis of lost-to-follow-up patients favors the study drug; and 6) other known molnupiravir trials were not presented in the primary study findings. As a result of methodological and statistical concerns, it seems that external trials, separate from those supported by the sponsoring company, are required to determine the utility of this drug.

Since the COVID-19 pandemic began, there has been a need for effective vaccines and treatments. The early vaccine trials allowed rapid development and deployment of highly effective vaccines, but treatments for COVID-19 have been slow to develop. The greatest successes among treatments have been those conducted in patients with advanced disease in hospitals.^1^ Much more elusive have been interventions for early treatment of COVID-19, when patients are not yet hospitalized.^1^ Clinical trials in this population are challenging because most patients will never be hospitalized regardless of interventions so identifying high-risk patients necessitates that clinical trials are conducted in settings with higher rates of co-morbid diseases for COVID-19 and lower vaccination rates.^2^

On October 1, 2021, Merck, in partnership with Ridgeback Biotherapeutics, issued a press release based on an interim analysis of ∼50% (*n* = 762) of patients recruited into an early-treatment phase 3 trial of high-risk COVID-19 patients.^3^ The company reported an approximate 50% reduction in hospitalization or death compared with placebo. This finding prompted the discontinuation of the clinical trial as a result of the reported superiority of the drug compared with placebo and, within about a week, discontinued recruitment at all other participating sites. What was unclear at the time was that upward of 90% of the planned sample of approximately 1,500 patients had already been recruited. The sponsor has maintained that the interim analysis should be considered the primary analysis for the study. When the study was finally published,^4^ the treatment effect had decreased to ∼30%, with CIs that overlap the point of nonsignificance when analyzed correctly. The drug has subsequently received US Food and Drug Administration (FDA) Emergency Use Authorization (EUA) and has already sold billions of dollars of pre-sales in the United States and internationally. There are, however, statistical and methodological concerns that may have resulted in overstatements of the treatment effect.

We address these issues with the Merck–Ridgeback MOVe-OUT trial evaluating molnupiravir.^4^ We focus on six points directly relevant to the clinical efficacy evaluation:

1. The authors and sponsors maintain that the interim analysis is the “formal efficacy” analysis, which is inconsistent with the protocol and primary statistical analysis plan.
2. Communication between sponsors and the Data Safety and Monitoring Board (DSMB) was insufficient to avoid inappropriate interim recommendations.
3. The treatment effects reverse when examining only the post-interim data, and are substantially attenuated when examining the full data.
4. The choice effect measure and statistical model for the primary analysis is incorrect.
5. The lost-to-follow-up analysis is unconventional. Conventional intention-to-treat analysis removes statistical significance.
6. Other known molnupiravir trials were not presented in the primary study findings.

First, in media statements and in the published trial,^3^^,^^4^ Merck consistently treated the presentation of the interim analysis of the MOVe-OUT trial as if it should be considered the primary efficacy analysis. The trial publication specifically states, “The planned interim analysis, in which efficacy results met the statistical criterion for superiority over placebo, represented the formal evaluation of efficacy for the trial; in accordance with the prespecified analysis plan, no additional statistical testing was performed for the primary efficacy end point.”^X,p^ In fact, the prespecified analysis plan states the opposite. Section IA4 on page 81 of the protocol states, “The purpose of this interim analysis is to allow for early stopping in the case of futility and to allow for the initiation of marketing authorization applications in the case of a positive efficacy finding. Given the expected rapid enrollment, there are no plans in Part 2 of the study to discontinue enrollment prior to the planned final sample size in the case of a positive efficacy outcome.”^ X,p^ There is no precedent in any form of clinical trial to change the primary efficacy analysis to only those data presented to the DSMB. Effectively changing the prespecified stopping time based on favorable early results has been described as a form of p-hacking.^5^ The correct analysis to emphasize in press releases and publications should be on all patients randomized.

Second, the processes by which exchange of information occurred between the study investigators and the DSMB were arguably not suitable for a relatively large-scale international COVID-19 trial during a rapidly evolving pandemic. The study protocol clearly states that the primary efficacy end point is the proportion of participants with either hospitalization (≥ 24 hours of acute care in a hospital or similar acute care facility, including emergency rooms or facilities created to address hospitalization needs during the COVID-19 pandemic) or death by day 29. The recommendation by the DSMB to stop the trial for efficacy (based on the first ∼50% of the data available) came at a time when ∼90% of the full sample had been recruited. The DSMB recommendation to stop early for efficacy and the investigators’ adherence to this recommendation places unnecessary pressure on stakeholders to consider the interim data in their interpretation of the efficacy of molnupiravir. The conventional randomized controlled trial setup, during which DSMBs take weeks to convene and review the interim data, is not conducive to COVID-19 because of the rapidly evolving nature of the pandemic. The MOVe-OUT trial is only one of many examples of undue reliance on positive interim analysis as investigators have rushed to the media without critical assessment of the potential consequences.

Third, at a key FDA EUA Antiviral Advisory Committee meeting, Merck did not present any exploratory analyses or other rationale for why the interim analysis showed a 50% reduction in hospitalization for molnupiravir, but then the second half of the study showed a trend favoring placebo and no difference in mortality (one event per group).^6^ Bias associated with interim analyses has been widely studied.^7^ A reversal of the treatment effects after an interim analyses seems unprecedented. In their interim analysis, Merck reported a ∼50% decrease in primary outcome events (28 of 385 versus 53 of 377) but this effect reversed after the interim analysis (20 of 324 in molnupiravir versus 15 of 322 in placebo). If we consider each separately, the likelihood that this occurred by chance alone is statistically unlikely (test of interaction, *P* < 0.01; Figure 1).

**Figure 1.**
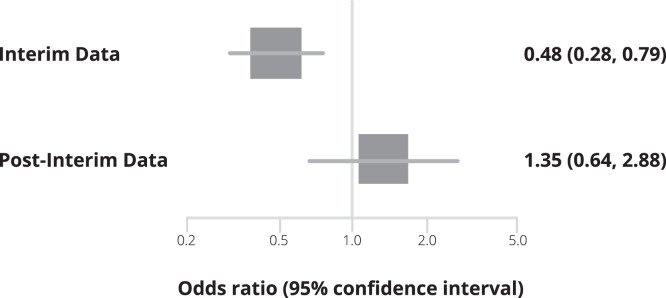
Subgroup forest plot of the interim data odds ratio versus the post-interim data (only) odds ratio with accompanying 95% CIs. Test-of-interaction subgroup effect yielded *P *< 0.01.

Fourth, at the FDA EUA advisory meeting, Merck presented their interim data across 10 countries, with no explanation for wide differences in outcomes among these countries.^6^ Point estimates of absolute risk differences (the primary analysis) varied from –19.6% for patients enrolled in Brazil to +9.1% for patients enrolled in Guatemala, with mutually exclusive CIs. When analyzing binary data, it is always recommended that both relative and absolute differences be presented, as both have serious limitations but complement each other well.^8^ Observed placebo risk also varied substantially across countries in the MOVe-OUT trial. When analyzing the full data, a relative effect measure such as a relative risk (RR) or odds ratio would be stable, whereas absolute risk differences do not generalize across countries. Relative effects are stable across varying control group risks. As evidence of the latter, a pooled 7% absolute risk reduction is meaningless for settings with less than 7% of events in the placebo group. A meta-analysis of the interim trial data using a random effects RR model that incorporates the heterogeneity of country effects, for example, finds a nonsignificant RR of 0.68 (95% CI, 0.46–1.00; see  Figure 2). Merck’s own analysis using a hazard ratio also finds a nonsignificant effect (RR, 0.69; 95% CI, 0.48–1.01).^4^

**Figure 2.**
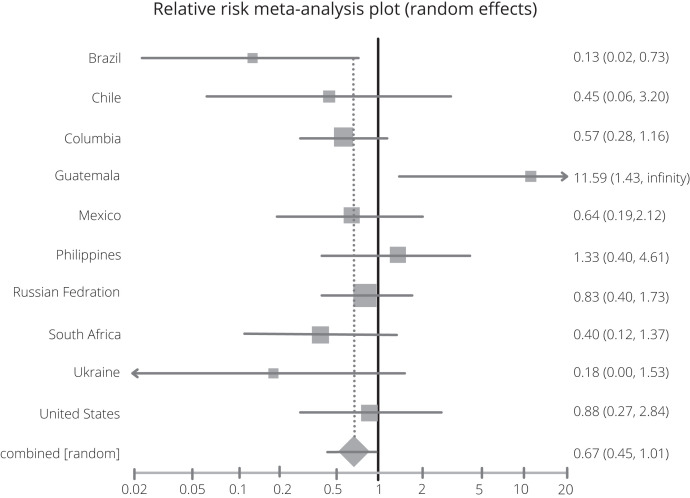
Relative risk random effects meta-analysis of country-specific aggregate data.

Fifth, the study protocol had prespecified that a lost-to-follow-up patient should be considered a primary event. This is an inappropriate assumption. The correct approach to assessing loss to follow-up is to use this assumption as a sensitivity analysis to determine whether the effects change in a meaningful way, the robustness of the data. Loss to follow-up occurred in one patient in the control group.^6^ In this case, the assumption of an event changed the interpretation importantly. If, instead, we assume no event, the current relative risk using the basic data would become nonsignificant (RR, 0.70; 95% CI, 0.49–1.00).

Sixth, and last, there have been at least three other molnupiravir trials that have been completed in similar populations but not published in peer-reviewed journals. The MOVe-IN trial, evaluating molnupiravir in hospitalized patients, showed no clinical benefits and was stopped by the trial ethics committee after slightly higher death rates were seen in the molnupiravir arm.^9^^,^^10^ Two other randomized trials of molnupiravir conducted by Indian pharmaceutical companies, Aurobindo and MSN Laboratories, enrolled more than 2,000 patients with moderate COVID-19 but showed no significant clinical benefits, according to the Indian regulatory authorities, and were discontinued.^11^ In these two trials, the definition of moderate COVID-19 included the criterion of blood oxygen levels between 90% and 93%, whereas the definition in the MOVe-OUT trial was “no lower than 93%.” A press release in July 2021 presented results from a positive interim analysis of another Indian trial of molnupiravir conducted by the Indian pharmaceutical company Hetero, with which Merck had entered into a non-exclusive licensing agreement.^12^^,^^13^ This analysis included 741 patients with mild COVID-19 from the total sample size of 1,218. This trial started recruitment on 25th May 2021, according to the Indian clinical trials registry, with results announced on July 9th, 2021. It is unclear how this trial could have recruited 741 patients, completed follow up, collected and analysed all the data within less than 7 weeks. This would be unprecedented for any randomised trial for COVID-19. In the media release, there was a statement that all adverse events were mild in severity, and none led to drug discontinuation. From a sample size of 741 patients, this seems very unlikely. By contrast, in the MOVe-OUT trial, 116 of 1,411 patients had at least one serious adverse event, and 30 of 1,411 patients discontinued randomized treatment because of adverse events. It is not clear why there should be no patients with these safety outcomes in one trial of molnupiravir and then an important number with the same outcomes in another trial. In the media release, there were 7/370 hospitalistions on molnupiravir (1.7%) versus 23/371 (6.2%) on control (*P* = 0.0027). In February 2022, results from all 1,218 patients were presented at a medical conference.^14^ As with the MOVe-OUT trial, there was no evidence of clinical benefit in the 477 additional patients recruited: 2/238 were hospitalized on molnupiravir, versus 3/239 in the control arm. Also, as with the MOVe-OUT trial, the hospitalization rate in the control arm fell significantly, from 23/371 (6.2%) in the first analysis to 3/239 (1.3%) for the patients randomized later. There is no explanation for this fall in hospitalization rate. There is no mention of these large, randomized trials in the publication of the MOVe-OUT trial.^4^ There was also no discussion of these clinical trials at the FDA EUA meeting. If all of these clinical trials were included in a meta-analysis, the summary effect would likely be nonsignificant, with wide CIs. The likelihood of this observation is suggested further by the fact that the All India Institute of Medical Sciences and the Indian Council for Medical Research (ICMR)–COVID-19 National Task Force recently (January 12, 2022) issued a second warning against molnupiravir based on review of data from the three outpatient trials mentioned earlier, stating that “harms far outweigh claimed benefits,”^15^ specifically among those at high risk of disease or older than 60 years.^16^ Other countries and organizations are currently (January 18, 2022) holding back, with France recently canceling their order molnupiravir,^17^ the United Kingdom only offering molnupiravir via the current Platform Adaptive Trial of Novel Antivirals for Early Treatment of COVID-19 In the Community (PANORAMIC) study (https://www.panoramictrial.org/), the European Medicines Evaluation Agency having not formally approved molnupiravir but only issued advice for countries considering its use,^18^ and the WHO having approved another drug (GSK’s sotrovimab) for mild infection based on clinical trial evidence generated around the same time as MOVe-OUT.

In the United Kingdom, the PANORAMIC study over 12,000 patients have been recruited to the PANORAMIC study as of March 3, 2022. The PANORAMIC trial is recruiting patients who are both vaccinated and unvaccinated (https://www.phctrials.ox.ac.uk/panoramic-trial). Given the inherent weaknesses in the MOVe-OUT study, there are several reasons regulatory authorities and other stakeholders should be wary when making important decisions regarding EUA or other meaningful endorsements of molnupiravir. Considering the strong clinical need in the early COVID-19 vulnerable population and the money at stake, the safeguards in place under the given time pressure unfortunately appear to have not been sufficient in this case. The MOVe-OUT trial is a single trial with several issues surrounding it. Full regulatory approval typically requires efficacy to be demonstrated in two separate randomized studies. Whether molnupiravir has an important role to play in this pandemic can now only be determined by well-conducted randomized controlled trials.
